# Machine learning developed a PI3K/Akt pathway-related signature for predicting prognosis and drug sensitivity in ovarian cancer

**DOI:** 10.18632/aging.205119

**Published:** 2023-10-17

**Authors:** Xiaofang Han, Liu Yang, Hui Tian, Yuanyuan Ji

**Affiliations:** 1 Core Laboratory, Shanxi Provincial People's Hospital (Fifth Hospital) of Shanxi Medical University, Taiyuan 030012, China

**Keywords:** immunotherapy, prognostic signature, ovarian cancer, machine learning, PI3K/Akt pathway

## Abstract

Background: Ovarian cancer is one of the deadliest malignancies among females, generally having a poor prognosis. The PI3K/Akt pathway plays a vital role in the oncogenesis and progression of many types of cancer. Limited studies have fully clarified the role of PI3K/Akt pathway in the prognosis of ovarian cancer and its correlation with drug sensitivity.

Methods: A prognostic PI3K/Akt pathway related signature (PRS) was constructed with 10 machine learning algorithms using TCGA, GSE14764, GSE26193, GSE26712, GSE63885 and GSE140082 datasets. Gaussian mixture and logistic regression were performed to identify the optimal models for classifying lymphatic and venous invasion.

Results: The optimal prognostic PRS developed by Lasso + survivalSVM algorithm acted as an independent risk factor for overall survival (OS) of ovarian cancer patients and had a good performance in evaluating OS rate of ovarian cancer patients. Significant correlation was obtained between PRS-based risk score and Immune score, ESTIMATE score, immune cells and cancer-related hallmarks. Low risk score indicated a lower immune escape score, TIDE score, and higher PD1&CTLA4 immunophenoscore in ovarian cancer. Moreover, PRS-based risk score acted as an indicator for drug sensitivity in the immunotherapy and chemotherapy of ovarian cancer patients.

Conclusions: All in all, our study developed a prognostic PRS showing powerful and good performance in predicting clinical outcome of ovarian cancer patients. PRS could serve as an indicator for drug sensitivity in the chemotherapy and immunotherapy.

## INTRODUCTION

Ovarian cancer is one of the deadliest malignancies among females. An estimated of 22,000 patients are diagnosed with this malignancy in the USA each year, ranking the eleventh most common malignancy and the fifth leading cause of cancer-related death among women [1]. The management of ovarian cancer has shifted from surgery alone to a multidisciplinary approach including surgery, chemotherapy, endocrine therapy, and immunotherapy. However, the 5-year overall survival (OS) rate for ovarian cancer patients are still less than 50% [2]. High recurrence and drug resistance remain the main reasons leading to the poor clinical outcomes for ovarian cancer patients [3]. Increasing evidences suggest immunotherapy as a promising modality for many malignancies, especially for advanced malignancies [4]. However, few drugs have been approved for the immunotherapy of ovarian cancer, which need to be further investigation. Moreover, limited biomarkers for monitoring the prognosis, drug sensitivity of immunotherapy and chemotherapy have been used for clinical application.

After being activated by other genes, Phosphatidylinositol-4,5-bisphosphate 3-kinase (PI3K) could result in protein kinase B (Akt) binding to the cell membrane in the PI3K/Akt signal transduction pathway [5]. As an intracellular signaling pathway, the PI3K/Akt pathway is correlated to cell cycle, proliferation, cancer and longevity [6]. Moreover, the PI3K/Akt pathway shows significant correlation with glycolysis, hypoxia, apoptosis, epithelial mesenchymal transition (EMT), tumor recurrence, and treatment resistance [5, 7–9]. Attacking the PI3K/Akt signaling pathway is suggested as one of therapeutic strategies in human cancer [10]. Some of genes in the PI3K/Akt signaling pathway have proved to be prognostic biomarkers in ovarian cancer, such KRAS [11]. However, limited studies have fully clarified the role of the PI3K/Akt signaling pathway related genes (PRGs) in the prognosis of ovarian cancer and its correlation with drug sensitivity.

After obtaining PRGs from Kyoto Encyclopedia of Genes and Genomes (KEGG) database, we then explored their expression and prognostic value in ovarian cancer. We then constructed an optimal PI3K/Akt signaling pathway related signature (PRS) for predicting the prognosis of ovarian cancer using 10 machine learning algorithms. Moreover, we also explored the correlation between PRS and tumor microenvironment as well as drug sensitivity in ovarian cancer. Our result may provide more evidences about the significant functions of the PI3K/Akt signaling pathway in the prognosis and drug sensitivity of cancers.

## MATERIALS AND METHODS

### Datasets sources

Supplementary Figure 1 showed the work flow of our study. Gene sets of PI3K/AKT pathway (n=354) were generated from KEGG PATHWAY Database (https://www.kegg.jp/kegg/pathway.html). RNA sequencing (RNA-seq) data of 374 ovarian cancer patients and 64 normal human ovarian samples were downloaded from TCGA database (https://portal.gdc.cancer.gov/repository) and GTEx database (https://xenabrowser.net/datapages/), respectively. Another five GEO datasets, including GSE14764 (n=80), GSE26193 (n=107), GSE26712 (n=185), GSE63885 (n=75) and GSE140082 (n=380), were used as testing cohorts for prognostic signature validation. The single cell expression data were isolated from GSE184880 dataset, including 5 normal tissues and 7 ovarian cancer tissues. Two immunotherapy cohorts (IMvigor210 (n=298) and GSE91061 (n=98) dataset) containing clinical information about the patients being treated with anti-PD-L1 and anti-CTLA4 agents were used to evaluate the performance of prognostic signature in predicting immunotherapy benefit.

### Integrative machine learning algorithms constructed an optimal PRS

After obtaining the differentially expressed genes (DEGs) with “limma” package using |LogFC| ≥ 1.5 as the cutoff, we then detected potential prognostic biomarkers for ovarian cancer among PRGs with univariate cox analysis (p<0.05). In order to develop an accurate and stable prognostic PRS for ovarian cancer, we then performed integrative analysis with 10 machine learning algorithms, including random survival forest (RSF), elastic network (Enet), Lasso, Ridge, stepwise Cox, CoxBoost, partial least squares regression for Cox (plsRcox), supervised principal components (SuperPC), generalized boosted regression modelling (GBM), and survival support vector machine (survival-SVM). Consistent with a previous study [12] and the link to the R scripts available on the Github website (https://github.com/Zaoqu-Liu/IRLS), we set the TCGA cohort as the training cohort and GEO cohorts as the testing cohort. Harrell’s concordance index (C-index) was calculated in all cohorts. The optimal prognostic PRG was regarded as the prognostic model with the highest average C-index. Based on the expression of genes in PRS and their corresponding coefficients, we then calculated the PRS score (risk score) of each OC patient. And OC patients were separated into high risk score and low risk score groups in each cohorts.

### Evaluation of the performance of PRS

The cut-off value was determined by the “surv_cutpoint” function of the R package “survminer”, which calculated statistics based on maximally selected rank statistics. Using “timeROC” package, we then generated time ROC curves, which could evaluate the performance of PRS in predicting the clinical outcome of ovarian cancer. We also randomly collected 54 prognostic signatures (Supplementary Table 1) that have developed for ovarian cancer and calculated their C-indexes using “rms” package. Moreover, univariate and multivariate cox analysis were performed to identify the risk factors among clinical characters and PRS for the prognosis of ovarian cancer. A predict nomogram based on PRS and clinical characters was constructed for ovarian cancer with “nomogramEx” R package.

### Immune infiltration analysis

Immunedeconv, an R package integrating 7 state-of-the-art algorithms, including CIBERSORT, MCPcounter, QUANTISEQ, XCELL, CIBERSORT-ABS, TIMER and EPIC [13], was used to explore the correlation between risk score and abundance of immune cells. The ESTIMATE algorithm was also used to calculate the Immune score and ESTIMATE score of OC patients by using the R package “estimate” [14].

### Drug sensitivity and gene set enrichment analyses

Two approaches, including immunophenoscore and Tumor Immune Dysfunction and Exclusion (TIDE) score, were suggested as reliable tools for predicting immunotherapy response. Immunophenoscore (IPS) of ovarian cancer cases were downloaded from The Cancer Immunome Atlas (TCIA, https://tcia.at/home). And the TIDE score and T cells exclusion scores of ovarian cancer cases were determined by TIDE (http://tide.dfci.harvard.edu). Gene sets of immune escape, immune surveillance and proliferation, were downloaded in previous publication (Supplementary Table 2) [15]. Hallmark gene sets were downloaded from Molecular Signatures Database (MSigDB). Using the R packages “clustersProfiler”, “enrichplot”, and “ggplot2”, we performed Gene Set Enrichment Analyses (GSEA) to improve our understanding of PRS related function and pathways. After downloading the drug sensitivity data of Genomics of Drug Sensitivity in Cancer (GDSC) (https://www.cancerrxgene.org/), we then calculated the half maximal inhibitory concentration (IC50) value of common drugs correlated with chemotherapy and endocrinotherapy of ovarian cancer cases with “oncoPredict” package.

### Gaussian mixture and logistic regression models for classifying lymphatic and venous invasion

Lymphatic and venous invasion were vital factors affecting the prognosis of ovarian cancer patients. We then identified the best model among PRS genes to classify lymphatic and venous invasion. Classification was conducted with model-based hierarchical agglomerative clustering based on the Gaussian finite mixture model. The PRS genes related clusters were classified by the Gaussian mixture model (GMM). Logistic regression analysis was performed to construct combined models to classify whether the patients had lymphatic and venous invasion.

### Single-cell RNA-seq analysis

ScRNA-seq data were under quality control prior to analysis. Cells with >25% of mitochondria-associated genes were filtered out. The top 2000 highly variable genes of each sample were normalized using the ScaleData function. RunPCA function was used to reduce the dimensionality of the PCA. Using “seurat” package, we then performed T-SNE analysis, which could map high dimensional cellular data into a two-dimensional space, grouping cells with similar expression patterns and separating those with different expression patterns. Cell types were determined by using SingleR method.

### Pseudotime analysis and cell-cell interaction analysis

Pseudotime analysis, also named cell trajectory analysis, could evaluate the evolutionary trajectory of apoptosis pathways and cell subtypes and infer the differentiation trajectory of certain cells during disease progression. We performed pseudotime analysis using “Monocle2” package. The pseudotime value was used by monocle to model the gene expression level as a nonlinear smooth pseudotime function to show change in gene expression with time. FDR < 0.05 was regarded as significant difference. We then explored the communication between immune cell subtypes using CellChat software, which contained the ligand-receptor information.

### ceRNA network

The upstream miRNA targets of PRS genes were predicted using several databases, including TargetScan, ENCORI, miRDB, RNAIter, TargetMiner, RNA22, miRwalk. And the upstream circRNAs interacting with miRNA were explored with StarBase 3.0.

### Statistical analysis

Statistical analyses were conducted with R software (version 4.2.1). Wilcoxon rank-sum test or student T test was performed to explore the difference between continuous variables. Pearson’s or Spearman's rank correlation analysis was conducted to analyze the correlations between two continuous variables. The two-sided log-rank test was used to test the difference in different Kaplan-Meier survival curves.

### Availability of data and materials

The analyzed data sets generated during the study were sourced from the TCGA database (https://portal.gdc.cancer.gov/repository) and GEO database (https://www.ncbi.nlm.nih.gov/geo).

## RESULTS

### The relationship of PRGs with ovarian cancer prognosis

**Figure 1:**
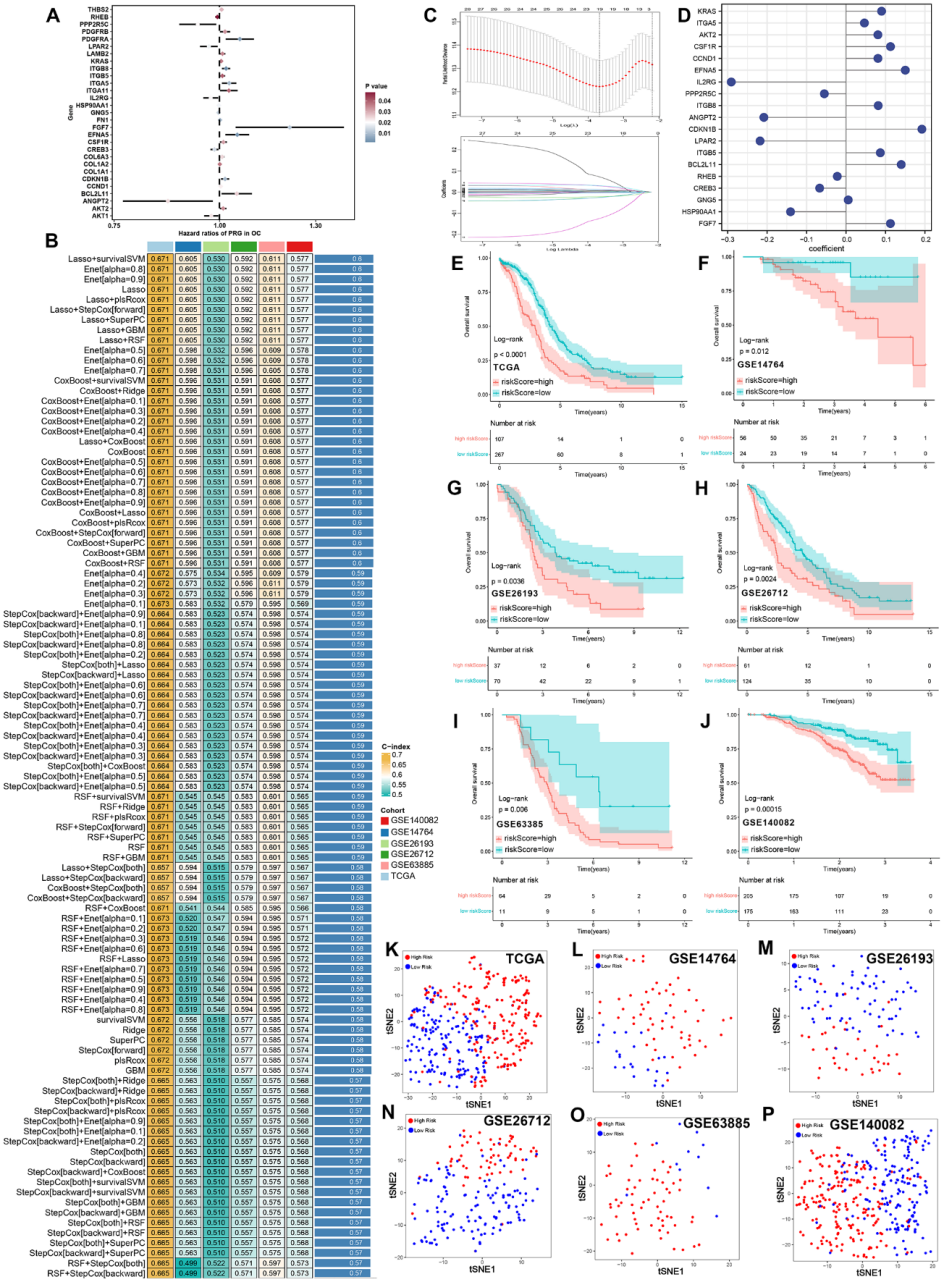
**Integrative machine learning analysis constructed a prognostic PI3K/Akt pathway related signature.** (**A**) Potential biomarker identified by univariate cox analysis. (**B**) The C-index of 101 kinds prognostic models constructed by 10 machine learning algorithms in training and testing cohort. (**C**) The determination of the optimal λ was obtained when the partial likelihood deviance reached the minimum value, and further generated Lasso coefficients of the most useful prognostic genes. (**D**) Coefficients of 19 genes finally obtained in survivalSVM regression. The survival curve of ovarian cancer with high and low risk score in TCGA (**E**), GSE14764 (**F**), GSE26193 (**G**), GSE26172 (**H**), GSE63885 (**I**) and GSE140082 (**J**) cohort. Cluster analysis of ovarian cancer cases with high and low risk score in TCGA (**K**), GSE14764 (**L**) GSE26193 (**M**), GSE26172 (**N**), GSE63885(**O**) and GSE140082 (**P**) cohort by using tSNE method.

As shown in Supplementary Figure 2A, a total of 174 DEGs were obtained in ovarian cancer with |LogFC| ≥ 1.5 as the cutoff (*p*<0.05). Supplementary Figure 2B showed the top 50 DEGs in ovarian cancer. Among these genes, 29 genes were significantly correlated with the prognosis of ovarian cancer (Figure 1A). To be more specific, ANGPT2, PPP2R5C, LPAR2, IL2RG, AKT1, CREB3, RHEB, GNG5, and HSP90AA1 were significantly correlated with a good clinical outcome in ovarian cancer (Figure 1A, HR<1, *p*<0.05). While COL1A1, COL1A2, FN1, CCND1, KRAS, THBS2, COL6A3, ITGB5, LAMB2, AKT2, CSF1R, PDGFRB, CDKN1B, ITGB8, ITGA11, ITGA5, BCL2L11, EFNA5, PDGFRA, and FGF7 were significantly correlated with a poor clinical outcome in ovarian cancer (Figure 1A, HR>1, *p*<0.05).

### Integrative machine learning algorithms developed an optimal prognostic PRS

Above 29 potential prognostic biomarkers were then subjected to an integrative procedure including 10 machine learning-based methods, with which we could develop an accurate and stable prognostic PRS. Aa result, a total of 101 kinds of prediction models were obtained, and their C-index of training cohort (TCGA) and testing cohort (GSE14764, GSE26193, GSE26712, GSE63885 and GSE140082) were shown in Figure 1B. We could see that the model constructed by Lasso + survivalSVM method was considered as the optimal model and they had a highest average C-index of 0.6 (Figure 1B). In the Lasso regression, the optimal λ was obtained when the partial likelihood deviance reached the minimum value based on the LOOCV framework (Figure 1C). A total of 19 genes with nonzero Lasso coefficients were submitted to survivalSVM, which identified a final set of 19 PRS and their coefficients were shown in Figure 1D. Using the cut-off value, we then divided into ovarian cancer cases into high and low risk groups based on their risk scores (PRS score). As expected, ovarian cancer patients with high risk score had a poor OS rate in TCGA (*p*<0.0001, Figure 1E), GSE14764 (*p*=0.012, Figure 1F), GSE26193 (*p*=0.0036, Figure 1G), GSE26712 (*p*=0.0024, Figure 1H), GSE63885 (*p*=0.006, Figure 1I) and GSE140082 (*p*=0.00015, Figure 1J) cohort. tSNE analysis about the classification of ovarian cancer cases into high- and low-risk groups of training cohort and testing cohort were shown in Figure 1K–1P. On the basis of above findings, we concluded that this PRS was capable of predicting the prognosis of ovarian cancer patients.

### Evaluation of the performance of PRS

**Figure 2:**
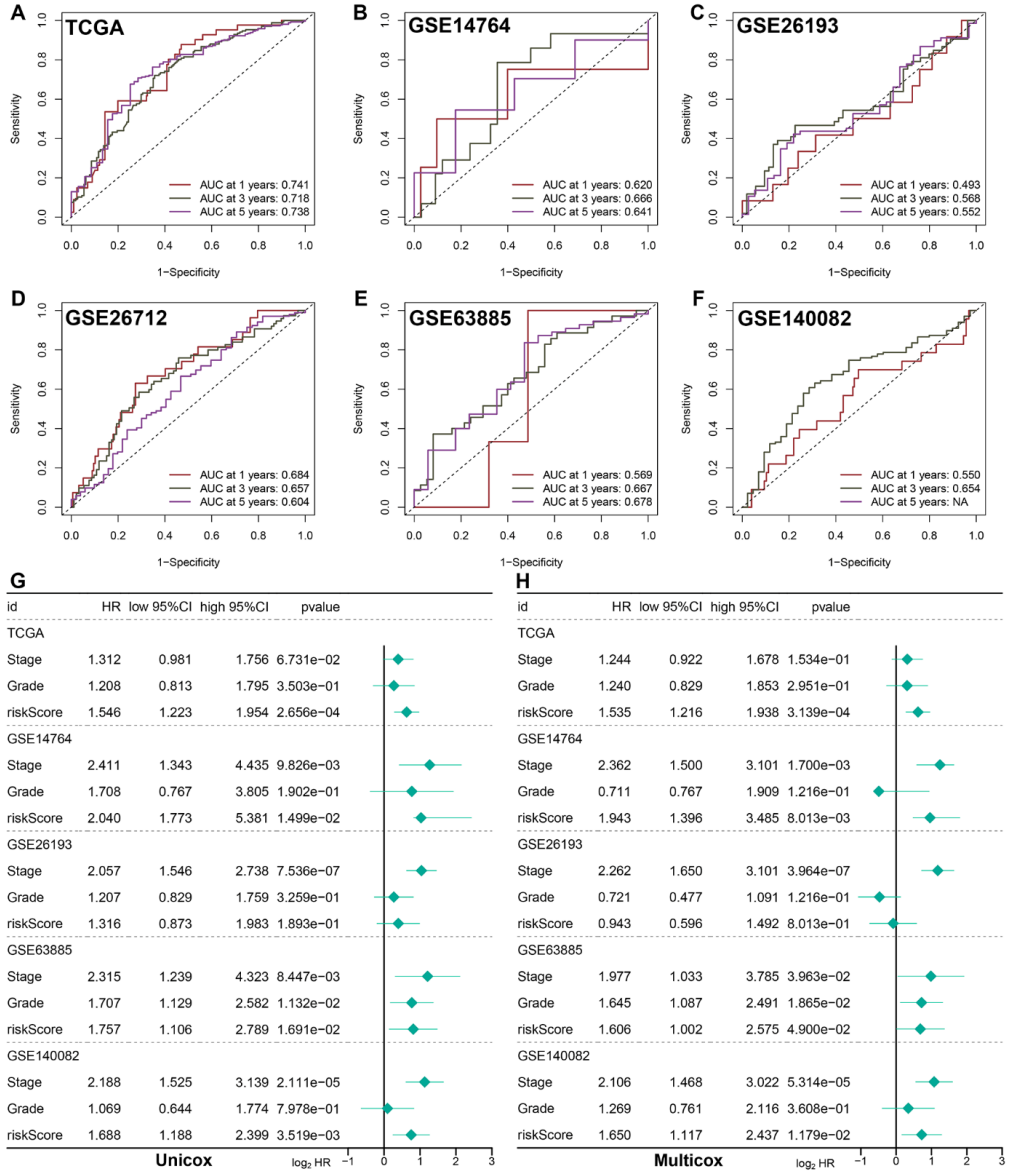
**Evaluation of the performance of prognostic PI3K/Akt pathway related signature (PRS).** ROC curve evaluated discrimination of PRS in predicting 1-, 3-, and 5-year OS rate of ovarian cancer in TCGA (**A**), GSE14764 (**B**), GSE26193 (**C**), GSE26172 (**D**), GSE63885 (**E**) and GSE140082 (**F**) cohort. (**G**, **H**) Univariate and multivariate cox regression analysis considering grade, stage and risk score in training and testing cohort.

ROC analysis was performed to evaluate the discrimination of PRS in training cohort and testing cohort, with 1-, 3-, and 5-year AUCs of 0.741, 0.718, and 0.738 in TCGA cohort (Figure 2A); 0.620, 0.666, and 0.641 in GSE14764 cohort (Figure 2B); 0.493, 0.568, and 0.552 in GSE26193 cohort (Figure 2C); 0.684, 0.657, and 0.604 in GSE26712 cohort (Figure 2D); 0.569, 0.667, and 0.678 in GSE63885 cohort (Figure 2E), respectively. In GSE140082 cohort, 1-, and 3-year AUCs were 0.550 and 0.640, respectively (Figure 2F). Further univariate and multivariate cox regression analysis demonstrated risk score as an independent risk factor for the prognosis of ovarian cancer patients in TCGA, GSE14764, GSE26193, GSE63885 and GSE140082 cohort (Figure 2G, 2H). We then compared the C-index of our PRS and 54 prognostic signatures that have been established for ovarian cancer. As shown in Supplementary Figure 3A, the C-index of the current PRS was higher than most of 54 random prognostic signatures. All in all, our PRS had a relatively good performance in predicting the clinical outcome of ovarian cancer patients. Considering PRS-based risk score, clinical stage and tumor grade, we then developed a nomogram for predicting the overall survival of ovarian cancer patients (Supplementary Figure 3B), which could help the clinicians evaluate the clinical outcome of ovarian cancer patients and make an appropriate follow-up project. Compared with the idea curve, nomogram-based calibration curves had a relative well predictive value in predicting the 1-, 3-, and 5-year OS rate (Supplementary Figure 3C).

### Development of the optimal model for evaluating the status of lymphatic and venous invasion

Lymphatic and venous invasion were vital factors affecting the prognosis of ovarian cancer. We then identified the optimal model among PRS genes to classify lymphatic and venous invasion. Logistic regression analysis was firstly performed to evaluate the association between the expression values of 19 genes in PRS and the AUC values that were screened the status of lymphatic and venous invasion. As a result, a total of 32767 formulas were generated from the logistic regression model. Furthermore, we used decisive GMM-based clustering, a very feasible approach with a good clustering performance [16, 17], to clustered gene sets into eight clusters (lymphatic invasion classifying) or nine clusters (venous invasion classifying) in our proposed algorithm. The cluster that had the highest AUC was considered as the best model to predict the status of lymphatic and venous invasion. As shown in Supplementary Figure 4B, the lymphatic invasion classifying model developed by 10 TRGs (CCND1, CDKN1B, CSF1R, EFNA5, FGF7, HSP90AA1, IL2RG, KRAS, LPAR2, RHEB) had a max AUC of 0.749 by the GMM classifier in one of the 32767 formulas. And venous invasion classifying developed by 11 TRGs (AKT2, CDKN1B, CREB3, CSF1R, EFNA5, FGF7, GNG5, HSP90AA1, ITGA5, ITGB8, RHEB) had a max AUC of 0.816 by the GMM classifier in one of the 32767 formulas (Supplementary Figure 4B).

### PRS showed significant correlation with cancer-related hallmark

**Figure 3:**
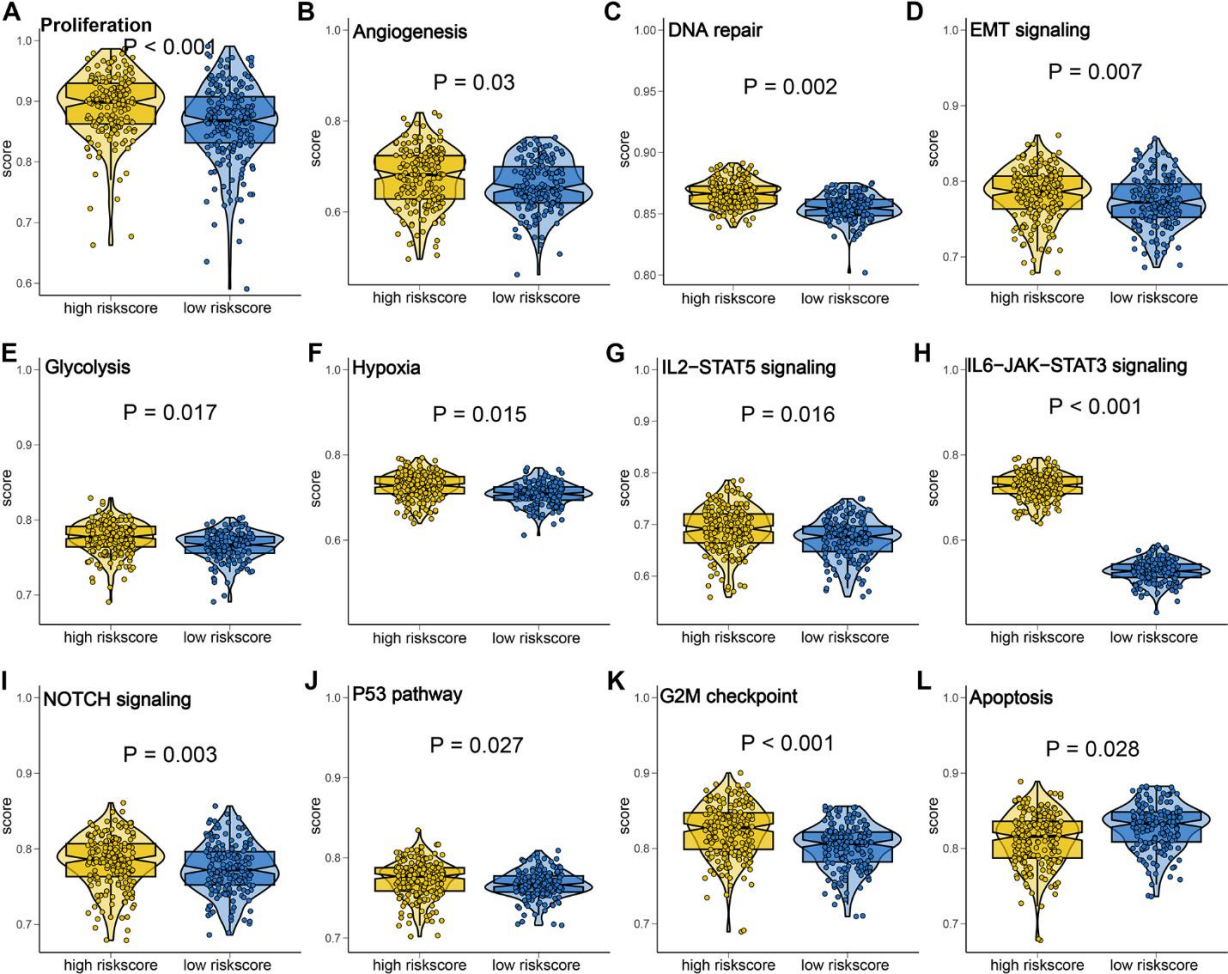
**Gene set enrichment analysis of PI3K/Akt pathway related signature (PRS).** The gene score of hallmark gene sets correlated with proliferation (**A**), angiogenesis score (**B**), DNA repair score (**C**), EMT signaling score (**D**), Glycolysis score (**E**), Hypoxia score (**F**), IL2-STAT5 signaling score (**G**), IL6-JAK-STAT3 signaling score (**H**), NOTCH signaling score (**I**), P53 pathway score (**J**), G2M checkpoint score (**K**) and apoptosis (**L**) in low and high risk score.

In order to clarify the vital role of PRS in ovarian cancer, we then perform GSEA analysis and explore PRS related biological process and internal connection in ovarian cancer. As expected, ovarian cancer with high risk score had a higher proliferation score, angiogenesis score, DNA repair score, EMT signaling score, Glycolysis score, and Hypoxia score (Figure 3A–3F, all *p*<0.05). Moreover, high risk score indicated a higher IL2-STAT5 signaling score, IL6-JAK-STAT3 signaling score, NOTCH signaling score, P53 pathway score, and G2M checkpoint score in ovarian cancer (Figure 3G–3K, all *p*<0.05). Interestingly, apoptosis score was significantly higher in ovarian cancer patients with low risk score (Figure 3L, *p*=0.028). Increasing evidences have highlighted vital role of macrophages M2/M1 proportion in tumor progression, prognosis and immunotherapy [18–20]. We found that high risk score indicated a higher level of macrophages M2/M1 proportion in TCGA, GSE26193, and GSE26712 cohort (Supplementary Figure 5A–5C, all *p*<0.05). These results implied that the activation of PI3K/ATK signaling participated in the tumor progression and shortened prognosis in ovarian cancer patients.

### The correlation between PRS and tumor microenvironment (TME) in ovarian cancer

**Figure 4:**
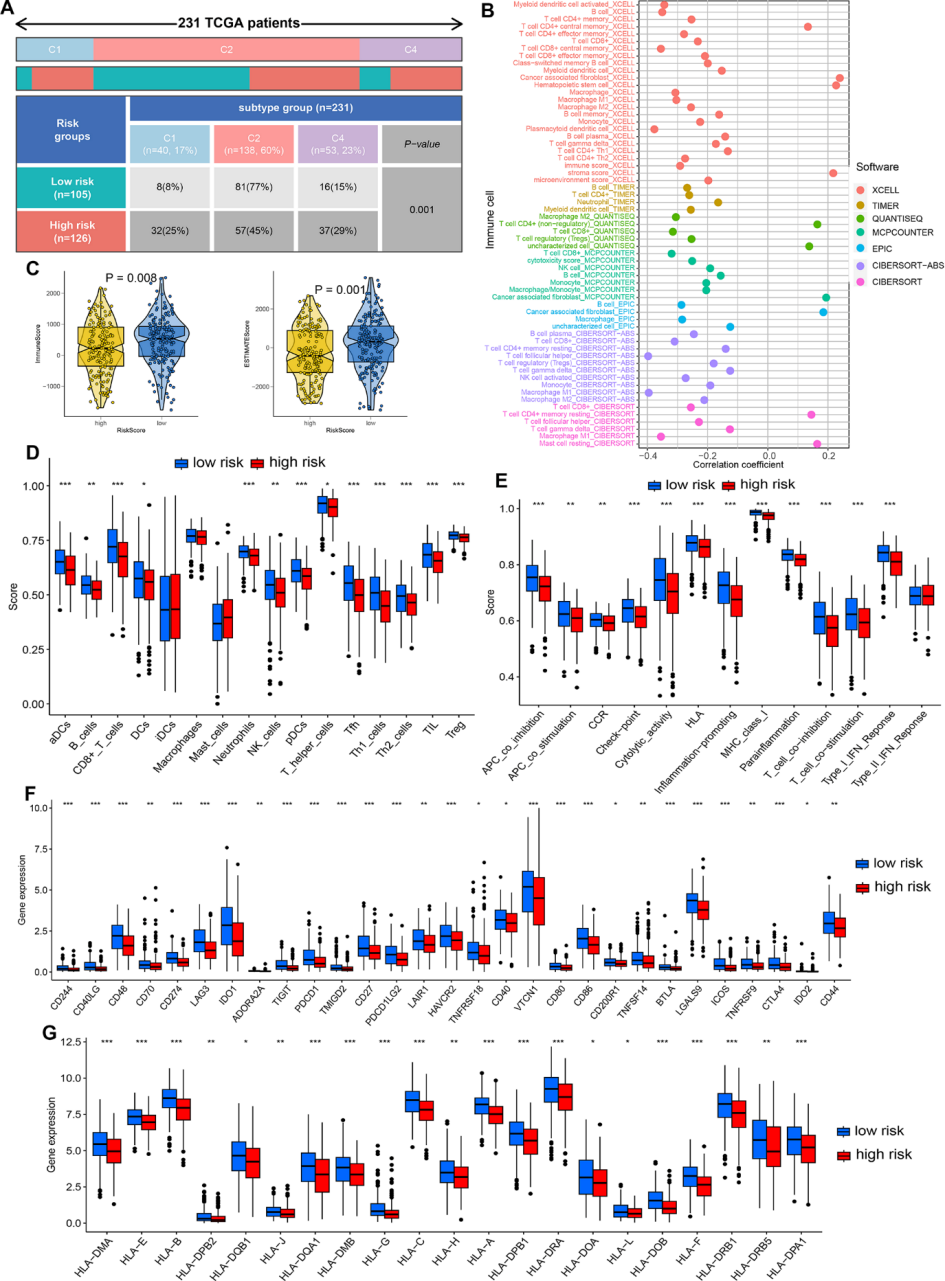
**Dissection of PI3K/Akt pathway related signature (PRS)-based tumor microenvironment (TME).** (**A**) Tumor immune landscape in ovarian cancer with high and low risk score. (**B**) Correlation between PRS and immune infiltration in ovarian cancer. (**C**) The TME score difference in different risk score group of ovarian cancer. The difference of the score of immune cells (**D**), immune-related functions (**E**), immune checkpoint (**F**), HLA-related genes (**G**) in different risk score group of ovarian cancer. *p<0.05, **p<0.01, ***p<0.001.

Tumor immune landscape could be clustered into six different types, including wound healing (C1), IFN-g dominant (C2), inflammatory (C3), lymphocyte depleted (C4), immunologically quiet (C5) and TGF-b dominant (C6) [21]. As shown in Figure 4A, C2 ranked most of TCGA ovarian cancer cases in both low and high risk group. However, the proportion of C4 in high risk group was significantly higher than that in low risk group (Figure 4A, *p*=0.001). With several algorithms including CIBERSORT, MCPcounter, QUANTISEQ, XCELL, CIBERSORT-ABS, TIMER and EPIC, we provided insights into the immune landscape in low and high risk groups to avoid inaccuracy and bias caused by the use of a single algorithm. Significant negative correlation was obtained between risk score and the abundance of most immune cells (Figure 4B). As expected, negative correlation was obtained between risk score and the abundance of most immune cells (Figure 4C, all *p*<0.05). As expected, low risk score indicated a higher ESTIMATE score and Immune score in ovarian cancer (Figure 4C, all *p*<0.05). In CIBERSORT algorithm, ovarian cancer with low risk score had a higher level of aDcs, B cells, CD8^+^ T cells, DCs, neutrophils, NK cells, pDCs, T helper cells, Tfh, Th1 cells, Th2 cells, TIL and Treg (Figure 4D). Moreover, low risk score was associated with a higher score of immune related functions in ovarian cancer, including APC_co_inhibition and stimulation, cytolytic activity, MHC cluss I, parainflammation, T cell co_inhibition, and type_I_IFN_reponse (Figure 4E, all *p*<0.05). Low risk score indicated a higher level of most of HLA-related genes (Figure 4F, *p*<0.05) and immune checkpoints (Figure 4G, *p*<0.05) in ovarian cancer patients. These results suggested that low risk score may be a relatively “hot” tumor phenotype compared with high risk score in ovarian cancer.

### Assessment of response to immunotherapy and chemotherapy between high and low risk score group

**Figure 5:**
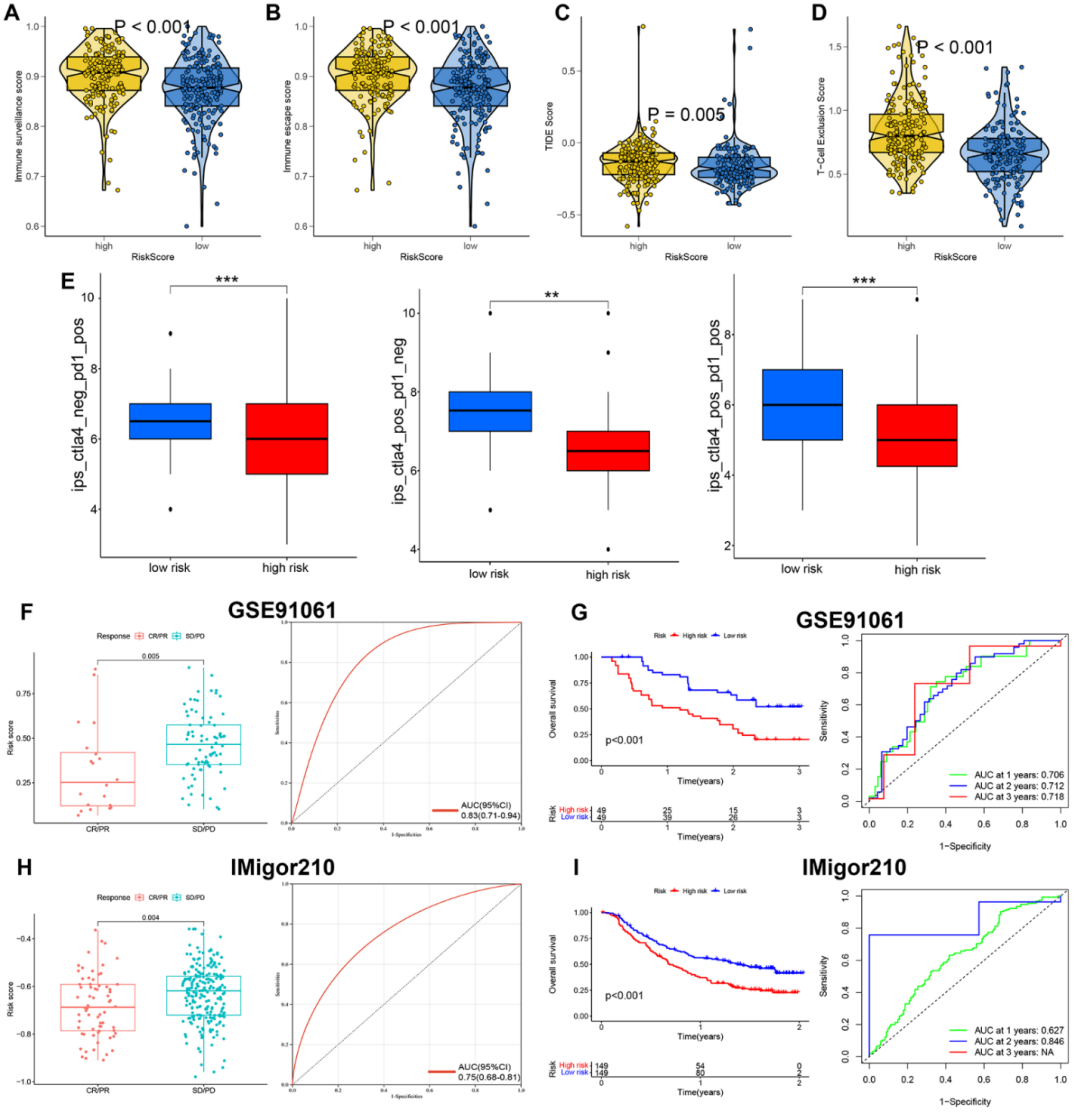
**PI3K/Akt pathway related signature (PRS)-based treatment strategy for ovarian cancer.** The level of immune surveillance score (**A**), immune escape (**B**), TIDE score (**C**), T cell exclusion score (**D**), and immunophenoscore (**E**) in ovarian cancer patients with high and low risk score. The risk score in CR/PR and SD/PD group and corresponding ROC curve in GSE91061 dataset (**F**). The OS curve and corresponding ROC curve in patients with high and low risk score in GSE91061 dataset (**G**). The risk score in CR/PR and SD/PD group and corresponding ROC curve in IMvigor210 dataset (**H**). The OS curve and corresponding ROC curve in patients with high and low risk score in IMvigor210 dataset (**I**). **p<0.01, ***p<0.001.

The above clarified the significant correlation between risk score and TME and the results suggested that ovarian cancer with low risk score may be a relatively “hot” tumor phenotype. Thus, we then explored whether risk score could predict the response to immunotherapy in ovarian cancer. As shown in Figure 5A, 5B, ovarian cancer with high risk score had a higher score of immune escape (Figure 5A, *p*<0.001) and immune surveillance (Figure 5B, *p*<0.001). Higher TIDE score and low IPS scores indicate higher immune escape potential and lower immunotherapy response rates. In our study, high risk score indicated a higher TIDE score (Figure 5C, p=0.005) and T cell exclusion score (Figure 5D, *p*<0.001) in ovarian cancer. IPS was a superior predictor of response to anti-CTLA-4 and anti-PD-1antibodies and high IPS indicated a better response to immunotherapy [22]. The results suggested that ovarian cancer with low risk score had a higher CTLA4 IPS, PD1 IPS and CTLA4/PD1 IPS (Figure 5E, all *p*<0.05). These results suggested that ovarian cancer patients with low risk score may be more sensitive to immunotherapy. In order to verify these results, we then used two immunotherapy cohorts, including IMvigor210 cohort and GSE91061 cohort. As shown in Figure 5F, patients in CR/PR group had a significant lower risk score, with an AUC of 0.83 in ROC curve (*p*=0.005). Interestingly, high risk score was associated with a poor OS rate, with 1-, 2-, and 3-year AUCs of 0.706, 0.712, and 0.718 (Figure 5G). Similar results were obtained in IMvigor210 dataset, which showed that CR/PR group indicated a lower risk score, with an AUC of 0.75 in ROC curve (Figure 5H, *p*=0.004). And patients with high risk score had a poor OS rate, with 1-, and 2- year AUCs of 0.627, 0.846 (Figure 5I, *p*<0.001). These evidences may suggest that ovarian cancer patients with low risk score may be more sensitive to immunotherapy and PRS may be an indicator for immunotherapy response. We then estimated the IC50 value of drugs correlated with chemotherapy and endocrinotherapy between high and low risk score group in order to guide clinical treatment in ovarian cancer. The result suggested that low risk score group tended to benefit from chemotherapy with 5-Fluorouracil, Cisplatin, Cyclophosphamide, Docetaxel, Epirubicin, Gemcitabine, Olaparib, Oxaliplatin, Topotecan, Tamoxifen, Erlotinib and Foretinib (Supplementary Figure 6A–6L, all *p*<0.05).

### The correlation between PRS and mutation landscape in ovarian cancer

As genetic mutation played a vital role in tumor genesis and progression. We then compared the difference of mutation landscape in high and low risk score group. Supplementary Figure 7A, 7B showed the mutation landscape of ovarian cancer in these two groups. Patients in low risk score group had a higher tumor mutational burden (TMB) score (Supplementary Figure 7C, *p*=0.001). Moreover, further prognostic analysis revealed that high TMB score and high risk score were associated with a poor OS rate (Supplementary Figure 7D, 7E, *p*<0.001).

### High-resolution scRNA-seq revealed the immune landscape of ovarian cancer

**Figure 6:**
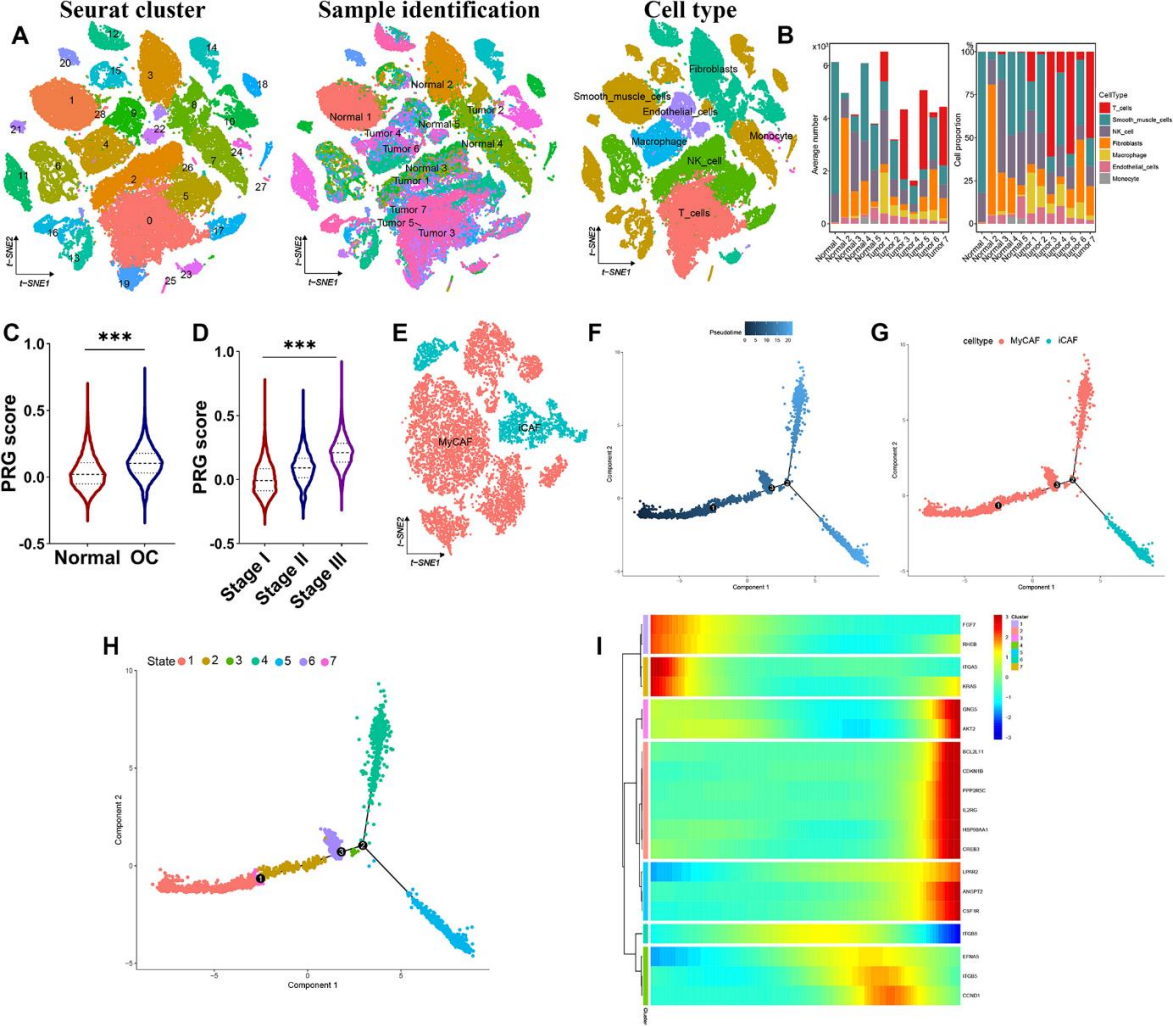
**High-resolution revealing immune landscape of ovarian cancer.** (**A**) t-SNE plot showing the identified cell types of all ovarian cancer and normal sample. (**B**) Fraction of cell types originating from each sample. (**C**, **D**) PI3K/Akt pathway related signature score in normal sample and ovarian cancer tissues. (**E**) Further sub-cell types of CAF cells. (**F**–**H**) Developmental trajectory of CAF cells inferred by monocle, colored by pseudotime, cell subtype and state. (**I**) Heatmap of the expression of PI3K/Akt pathway related signature genes in the developmental trajectory of CAF cells state. ***p<0.001.

A total of 5 normal tissues and 7 ovarian cancer tissues were analyzed to characterize the immune landscape in ovarian cancer. As shown in Figure 6A, all the cells in these tissues could be clustered into 7 subtypes, including T cells, Smooth muscle cells, NK cell, Fibroblasts, Macrophage, NK cell, and Endothelial cells and Monocyte. We then explored the fraction of different immune cell types in each sample, revealing that different immune cell types varied significantly among different samples (Figure 6B). To be specific, T cells were prevalent in tumor tissues while smooth muscle cells were predominant in normal tissues (Figure 6B). Compared with normal tissues, ovarian tissues had a high PRG score (Figure 6C). With the progression of ovarian cancer, the PRG score was increasing (Figure 6D). These results further revealed that the activation of PI3K/ATK signaling participated in the tumor progression and shortened prognosis in ovarian cancer patients. As cancer-associated Fibroblast (CAF) plays a vital role in tumor progression. We then collected Fibroblast in tumor for further analysis. Using t-SNE analysis, CAF were categorized as myCAF and iCAF cells (Figure 6E). Further analysis revealed a different trajectory between different pseudotime, state, and cell subtypes in the development trajectory analyses of CAF cells (Figure 6F–6H). Figure 6I revealed the expression of PRG in different state of CAF cells.

### Cell communication network analysis in ovarian cancer

We studied the signaling pathway that allowed multiple cells to interact with each other during tumorigenesis in order to illustrate how these cells regulate tumorigenesis. The number and strength of interactions were shown in Supplementary Figure 8A. We then focus on interaction number and weights/strength of CAF cells with other cell types. The CAF cells were strongly linked with smooth muscle cell, endothelial cells and Monocyte (Supplementary Figure 8B). Further analysis revealed that PRS genes were involved in FGF signaling network in ovarian cancer. FGF signaling could be activated CAF-CAF interaction (Supplementary Figure 8C, 8D). In FGF signaling, FGF7 and FGFR1 was the ligand and receptor, respectively (Supplementary Figure 8E). FGF7 from CAFs may interact with those smooth muscle cell, endothelial cells and Monocyte have FGFR1 expression (Supplementary Figure 8F).

### ceRNA network

The above results revealed that FGF7 may play a vital role in the progression of ovarian cancer. We then explore their upstream targets using several databases. As showed in Supplementary Figure 9A, miR-132-3p, miR-195-5p and miR-18b-5p were suggested as potential miRNA targets of FGF7 based on the result of TargetScan, ENCORI, miRDB, RNAIter, TargetMiner, RNA22, miRwalk. We then explored the upstream circRNAs interacting with miR-132-3p, miR-195-5p and miR-18b-5p. As a result, a total of 91 circRNAs were obtained. The circRNA-miRNA-mRNA network was showed in Supplementary Figure 9B.

## DISCUSSION

Ovarian cancer is one of the most common malignancies among women and its prognosis is poor [23]. Increasing evidences suggest the vital role of the PI3K/Akt pathway in cell cycle, proliferation, cancer, longevity, prognosis and therapy of cancer [24–27]. Moreover, the PI3K/Akt pathway showed significant correlation with glycolysis, hypoxia, apoptosis, epithelial mesenchymal transition (EMT), tumor recurrence, and treatment resistance [5, 7–9]. However, few studies have comprehensively and systematically described the characteristics of the PI3K/Akt pathway related genes in ovarian cancer.

Differentially expressed analysis identified 174 differentially expressed genes (DEGs) of the PI3K/Akt pathway in ovarian cancer. Among these DEGs, a total of 29 genes were significantly correlated with the prognosis of ovarian cancer. We then performed an integrative pipeline including 10 machine learning algorithms, which could develop a powerful prognostic PRS. As a result, the model constructed by Lasso + survivalSVM method was considered as the optimal model with the highest average C-index of 0.6. Moreover, further analysis suggested TRS as an independent risk factor for the overall survival of ovarian cancer patients. Actually, many prognostic models had been constructed for ovarian cancer, including Immune-related LncRNA signature [28], glycometabolism-related signature [29], oxidative stress-related signature [30], transcription factors-based signature [31], ferroptosis-related signature [32] and invasion-related gene signature [33]. In order to compare the performance of these prognostic signatures in evaluating the prognosis of ovarian cancer, we then calculated their C-index. Interestingly, the C-index of PRS was higher than most of other signatures, suggesting that our PRS may have a better performance in predicting the prognosis of ovarian cancer patients.

Immune cell recruitment to the tumor microenvironment is a promising therapeutic strategy, even in aggressive tumors. Increasing evidences have highlighted the vital role of the PI3K/ATK pathway in clinical management of cancer [34, 35]. Conventional chemotherapeutics exert tumor-suppressive effects mainly by inducing the release of DMAPs from cancer cells, activating the presentation of DC cells, thus activating CD8^+^ T cells to kill cancer cells. The efficacy of these immunotherapy agents and their correlation with the PI3K/ATK pathway [36, 37]. In order to further clarify the role of PRS in TME, we applied several algorithms to explore the underlying association of PRS with immune infiltration. As a result, some immune cells, including T cells, B cells, myeloid dendritic cells, and neutrophils, were more active in PRS-based low risk score group. Moreover, some cancer-related hallmarks were more active in PRS-based high risk score group, including proliferation, angiogenesis, DNA repair, EMT signaling, Glycolysis, Hypoxia, NOTCH signaling, and P53 pathway. These evidences identified that PRS might be involved in the development of ovarian cancer by regulating tumor immunity.

Targeting immune checkpoint molecules can activate anti-tumor immunity to help clear tumors [38].

Immunotherapy has revolutionized the situation of patients with unresectable cancers [39]. Until now, limited effective biomarkers for predicting immunotherapy efficacy have been used clinically, though some biomarkers, including PD-1, PD-L1, MSI, TMB etc., have been identified. Since significant correlation was obtained between PRS and immune infiltration, we further explored the role of PRS in predicting immunotherapy efficacy. As an increased TIDE score indicates a greater likelihood of immune escape and less effectiveness of ICI treatment [40]. IPS was a superior predictor of response to anti-CTLA-4 and anti-PD-1 antibodies and high IPS indicated a better response to immunotherapy [22]. In our study, ovarian cancer with high risk score had a higher immune escape score, higher immune surveillance score, higher TIDE, lower TMB and lower IPS scores. It seems reasonable to assume that patients with PRS-based low risk score benefit more from immunotherapy in terms of the treatment strategies for ovarian cancer. Further studies suggested that the risk score in CR/PR group was lower than that in SD/PD group in GSE91061 and IMvigor210 dataset, which further verifies our results.

Chemotherapy and endocrine therapy were vital therapeutic measures of ovarian cancer. Chemoresistance was one of the most reasons leading to treatment failure of ovarian cancer [41]. The result suggested that low risk group tended to benefit from chemotherapy and endocrine therapy with 5-Fluorouracil, Cisplatin, Cyclophosphamide, Docetaxel, Epirubicin, Gemcitabine, Olaparib, Oxaliplatin, Topotecan, Tamoxifen, Erlotinib and Foretinib, demonstrating that PRS was an indicator for the chemotherapy response of ovarian cancer.

Some limitations and shortcomings remain in our study. All data are obtained from public databases and it would be better to validate this prognostic model using clinical data. Moreover, the mechanism of PRS related genes in the progression of ovarian cancer remains unknown. A more in-depth investigation of these genes in ovarian cancer development will be undertaken *in vivo* or *in vitro*.

## CONCLUSIONS

All in all, our study developed a prognostic PRS showing powerful and good performance in predicting the clinical outcome of ovarian cancer patients. PRS could serve as an indicator for drug sensitivity in the chemotherapy and immunotherapy.

## Supplementary Material

Supplementary Figures

Supplementary Table 1

Supplementary Table 2
