# Supplementary material for: Machine learning developed a PI3K/Akt pathway-related signature for predicting prognosis and drug sensitivity in ovarian cancer
Source: Aging (Albany NY). 2023 Oct 17;15(20):11162–83. doi: 10.18632/aging.205119 (PMC10637788; doi:10.18632/aging.205119)
Supplement: Supplementary Figures [file aging-15-205119-s001.pdf]

SUPPLEMENTARY FIGURES

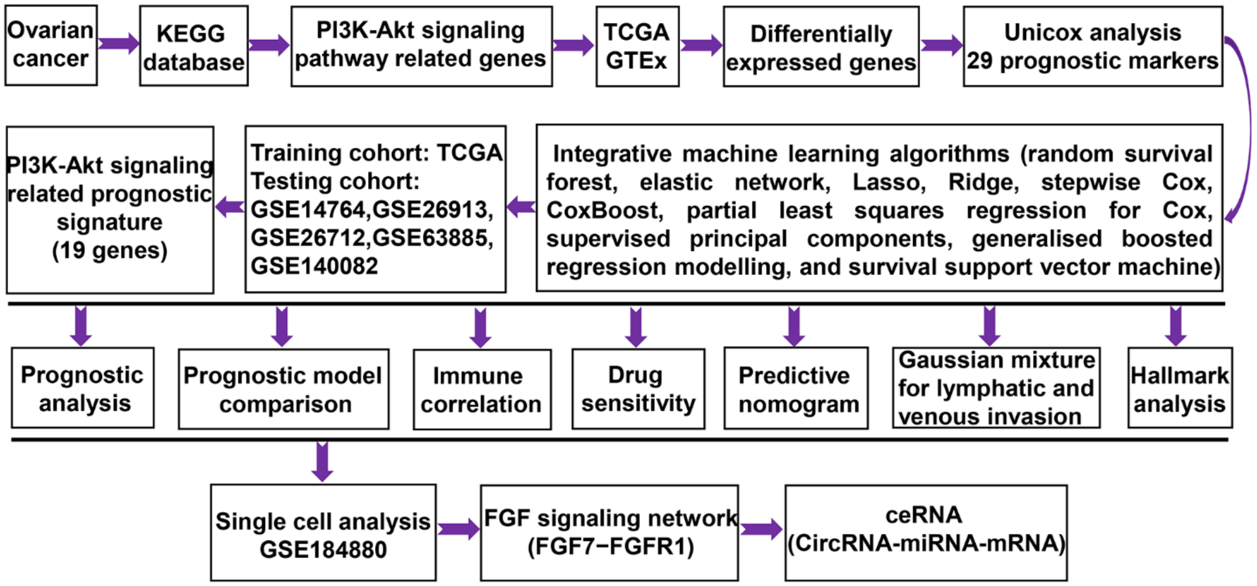

Supplementary Figure 1. Study workflow.

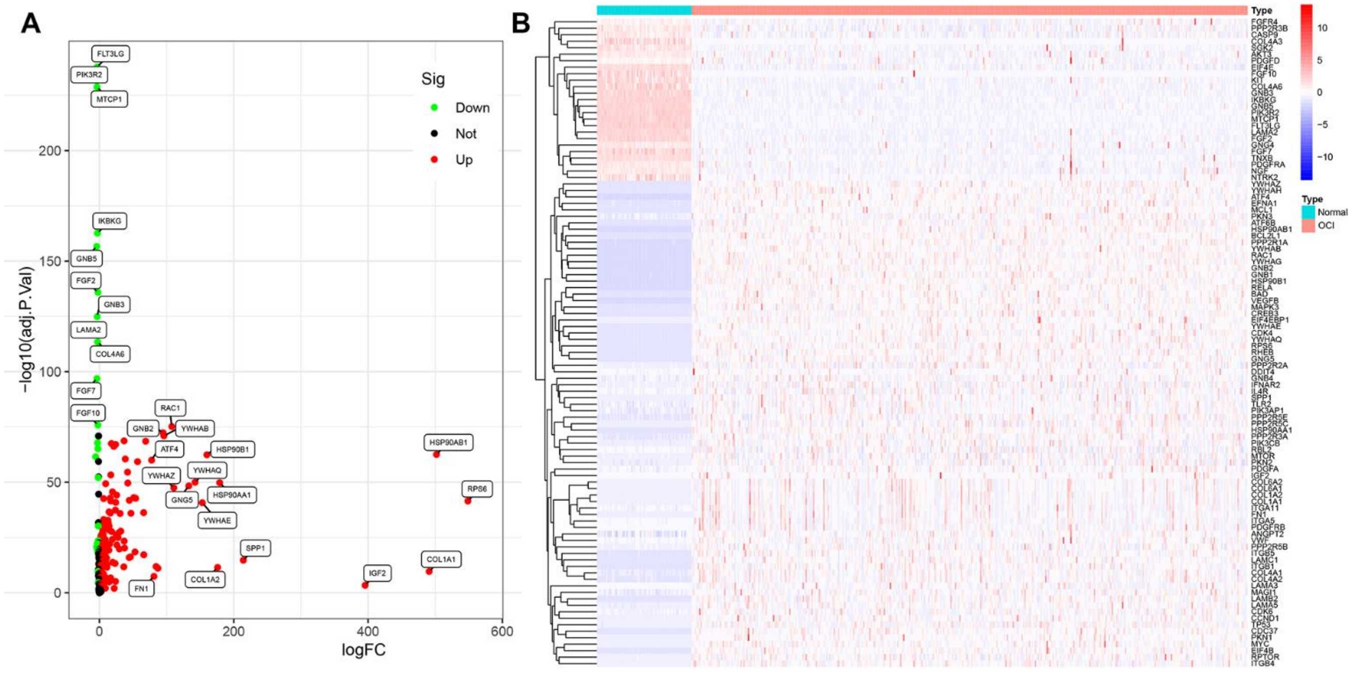

Supplementary Figure 2. Differentially expressed genes between ovarian cancer and normal tissues. (**A**) Volcanic map showed all differentially expressed genes in ovarian cancer among PI3K/Akt pathway related genes. (**B**) Heatmap showed top 50 differentially expressed genes in ovarian cancer among PI3K/Akt pathway related genes.

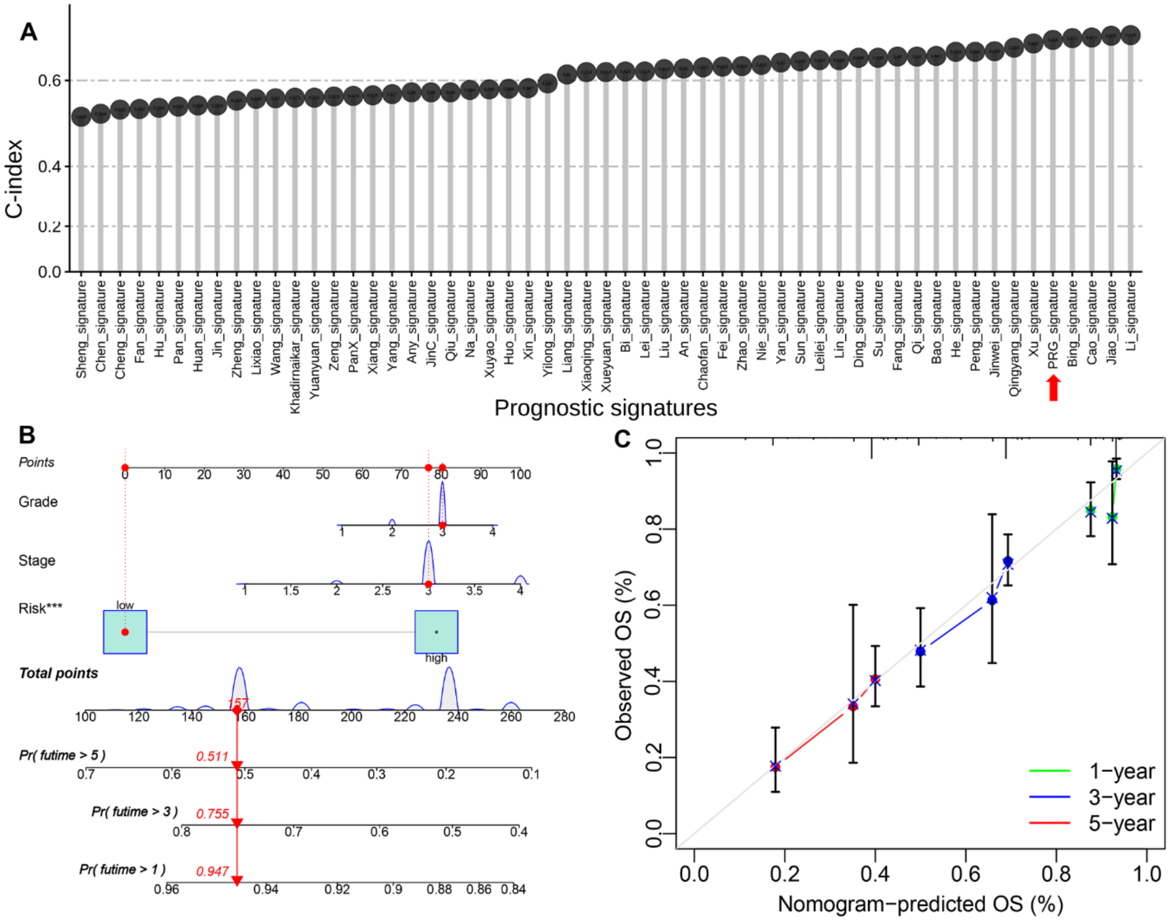

Supplementary Figure 3. The performance of prognostic PI3K/Akt pathway related signature (PRS) in predicting the prognosis of ovarian cancer. (**A**) C-index of PRS and other established signatures evaluated the prognosis of ovarian cancer patients. (**B**, **C**) Prediction nomogram for predicting the 1-, 3-, and 5-year OS rate of ovarian cancer.

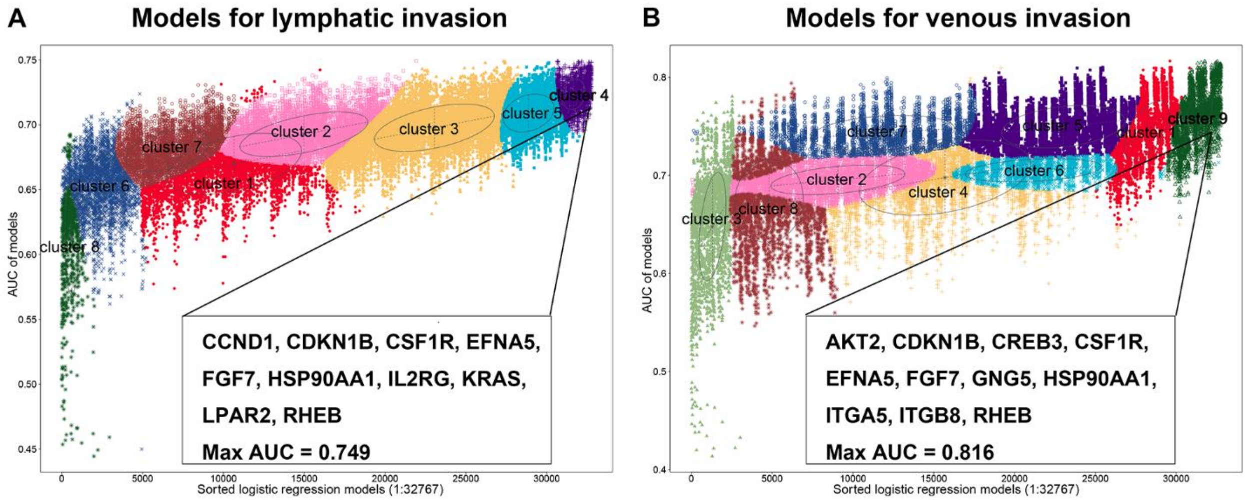

Supplementary Figure 4. The pattern of AUC and logistic regression models was based on Gaussian finite mixture models for classifying lymphatic and venous invasion. The pattern of the logistic regression model correlated with the AUC scores and was identified by a Gaussian mixture for classifying lymphatic (**A**) and venous invasion (**B**).

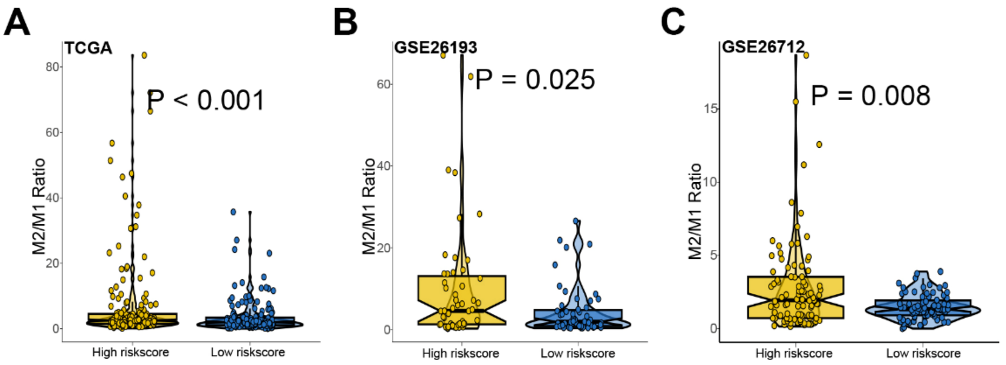

Supplementary Figure 5. The level of macrophages M2/M1 proportion in ovarian cancer patients with high and low risk score in TCGA (**A**), GSE26193 (**B**) and GSE26712 (**C**) cohort.

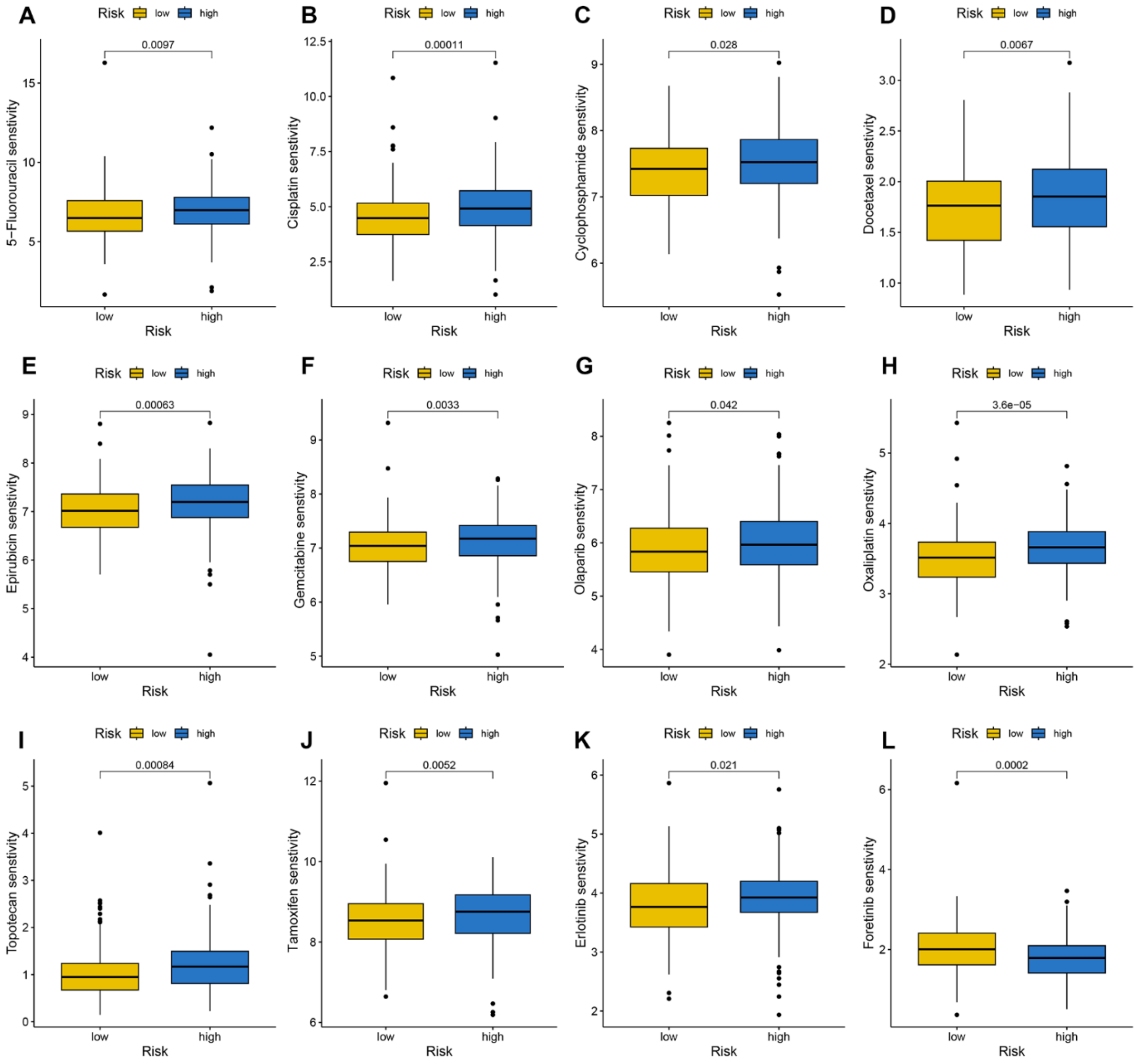

Supplementary Figure 6. PI3K/Akt pathway related signature (PRS)-based treatment strategy for ovarian cancer. The IC50 values of 5-Fluorouracil (**A**), Cisplatin (**B**), Cyclophosphamide (**C**), Docetaxel (**D**), Epirubicin (**E**), Gemcitabine (**F**), Olaparib (**G**), Oxaliplatin (**H**), Topotecan (**I**), Tamoxifen (**J**), Erlotinib (**K**), and Foretinib (**L**) in different risk score group of ovarian cancer.

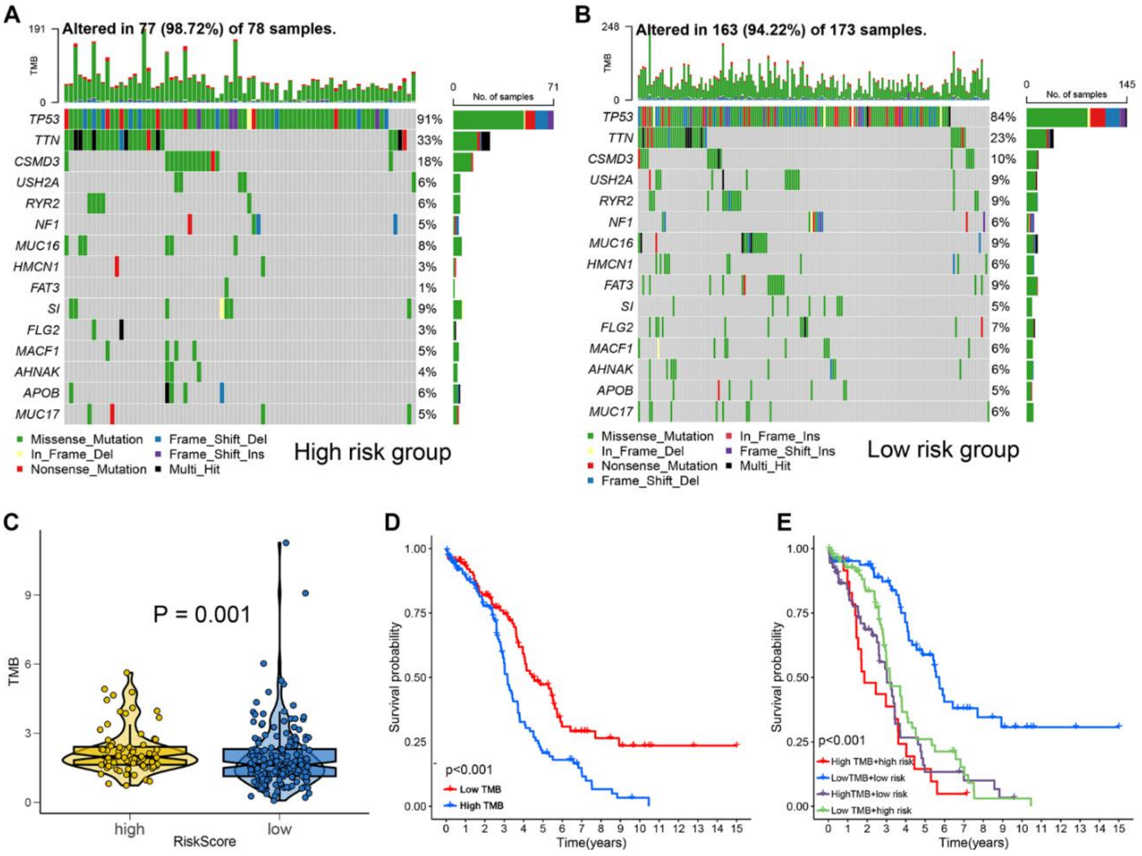

Supplementary Figure 7. Dissection of PI3K/Akt pathway-related signature (PRS)-based genetic mutation. (**A**, **B**) Genetic landscape in different risk score group of ovarian cancer. (**C**) The tumor mutational burden score in different risk score of ovarian cancer. (**D**, **E**) The overall survival curve in ovarian cancer patients with different tumor mutational burden and risk score.

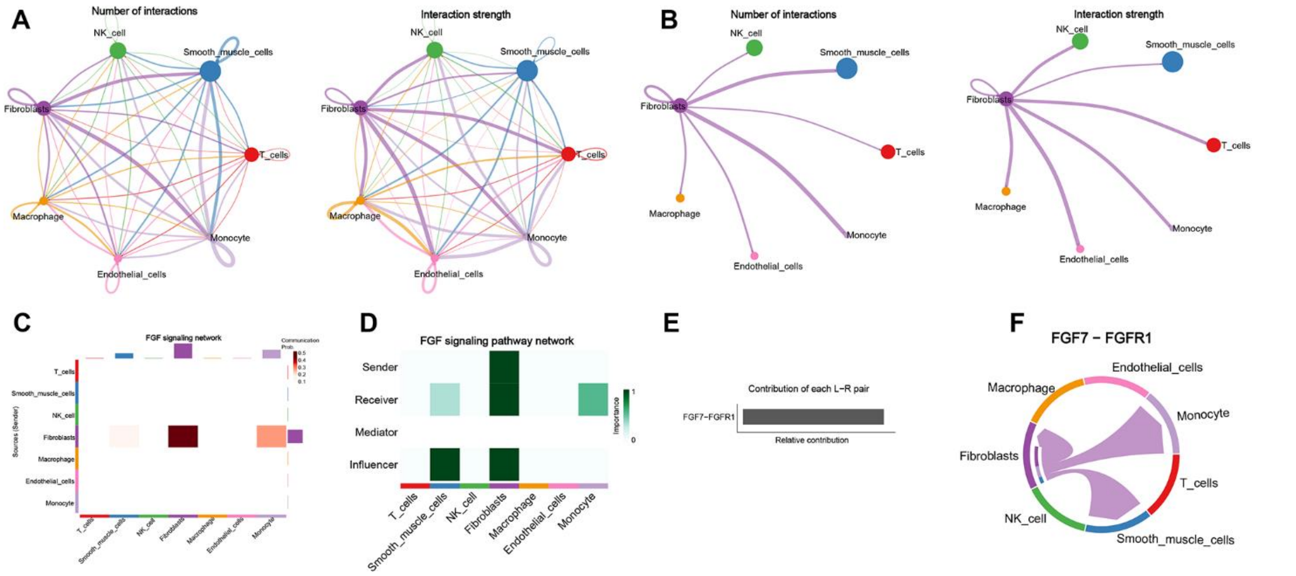

Supplementary Figure 8. Cell communication network analysis in ovarian cancer. (**A**) Chord plot showing the number and weights/strength of interactions among all cell types. (**B**) The interaction number and weights/strength of CAF cells with other cell types. (**C**, **D**) The interaction of all cell types in PI3K/Akt pathway related signature (PRS) related FGF signaling network. (**E**) The sender and receiver pair in FGF signaling network. (**F**) Chart showing inferred intercellular communication network of FGF signaling pathway in all cell types.

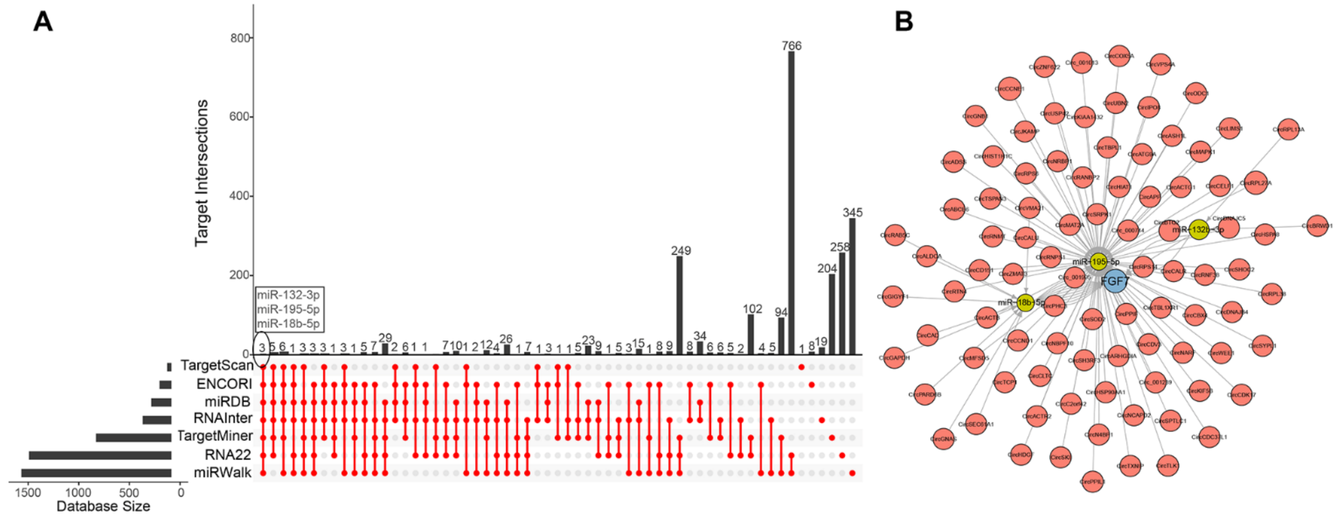

Supplementary Figure 9. ceRNA network associated with hub gene FGF7. (**A**) miRNA target of FGF7 predicted by TargetScan, ENCORI, miRDB, RNAIter, TargetMiner, RNA22, miRwalk. (**B**) circRNA interacting with miRNA preded by StarBase 3.0 and the circRNA-miRNA-miRNA.
