# Supplementary material for: Machine learning developed a PI3K/Akt pathway-related signature for predicting prognosis and drug sensitivity in ovarian cancer
Source: Aging (Albany NY). 2023 Oct 17;15(20):11162–83. doi: 10.18632/aging.205119 (PMC10637788; doi:10.18632/aging.205119)
Supplement: Supplementary Table 1 [file aging-15-205119-s002.pdf]

SUPPLEMENTARY TABLE

Supplementary Table 1. Other models have been established for ovarian cancer.

Signature	Title	PMID
An	Development of a Novel Autophagy-related Prognostic Signature for Serous Ovarian Cancer	30410611
Any	The Comprehensive Analysis of Interferon-Related Prognostic Signature with regard to Immune Features in Ovarian Cancer.	35769811
Bao	Novel gene signatures for prognosis prediction in ovarian cancer	32666642
Bi	Establishment of a novel glycolysis-related prognostic gene signature for ovarian cancer and its relationships with immune infiltration of the tumor microenvironment	34496868
Bing	Novel Model for Comprehensive Assessment of Robust Prognostic Gene Signature in Ovarian Cancer Across Different Independent Datasets	31681404
Cao	Development of a multi-gene-based immune prognostic signature in ovarian cancer	33509250
Chaofan	Establishment and validation of an RNA binding protein-associated prognostic model for ovarian cancer	33550985
Chen	Integrating cell cycle score for precise risk stratification in ovarian cancer	36061171
Cheng	Construction and validation of a transcription factors-based prognostic signature for ovarian cancer.	35227285
Ding	Construction of a new tumor immunity-related signature to assess and classify the prognostic risk of ovarian cancer	33154188
Fan	A newly defined risk signature, consisting of three m6A RNA methylation regulators, predicts the prognosis of ovarian cancer	32950970
Fang	Establishment, immunological analysis, and drug prediction of a prognostic signature of ovarian cancer related to histone acetylation	36172179
Fei	Construction autophagy-related prognostic risk signature to facilitate survival prediction, individual treatment and biomarker excavation of epithelial ovarian cancer patients.	33676525
He	Development of a novel transcription factors-related prognostic signature for serous ovarian cancer	33785763
Hu	Identification of a five-gene signature of the RGS gene family with prognostic value in ovarian cancer.	33845140
Huan	Integrated Analysis of Ferroptosis-Related Biomarker Signatures to Improve the Diagnosis and Prognosis Prediction of Ovarian Cancer	35071242
Huo	Identification of a Prognostic Signature for Ovarian Cancer Based on the Microenvironment Genes	34054929
Jiao	N6-Methyladenosine-Related RNA Signature Predicting the Prognosis of Ovarian Cancer.	34137363
Jin	A panel of three oxidative stress-related genes predicts overall survival in ovarian cancer patients received platinum-based chemotherapy	29910195
JinC	A 2-Protein Signature Predicting Clinical Outcome in High-Grade Serous Ovarian Cancer	28976449
Jinwei	Identification and verification of a ten-gene signature predicting overall survival for ovarian cancer	32805252
Khadirnaikar	Development and validation of an immune prognostic signature for ovarian carcinoma	32794637
Lei	Identification of an energy metabolism-related gene signature in ovarian cancer prognosis	32186777
Leilei	Establishment and validation of a novel invasion-related gene signature for predicting the prognosis of ovarian cancer.	35292033
Li	Identification and validation of a gene-based signature reveals SLC25A10 as a novel prognostic indicator for patients with ovarian cancer	36114504
Liang	A Novel Glycosyltransferase-Related Gene Signature for Overall Survival Prediction in Patients with Ovarian Cancer	34992448
Lin	A methylation-driven genes prognostic signature and the immune microenvironment in epithelial ovarian cancer.	35783253
Liu	Construction and validation of a novel aging-related gene signature and prognostic nomogram for predicting the overall survival in ovarian cancer.	34825509
Lixiao	Construction and Validation of a Novel Glycometabolism-Related Gene Signature Predicting Survival in Patients With Ovarian Cancer.	33281878
Na	Comprehensive Analysis of Tumor Microenvironment Identified Prognostic Immune-Related Gene Signature in Ovarian Cancer	33679883
Nie	Prognostic signature of ovarian cancer based on 14 tumor microenvironment-related genes	34260536
Pan	A Potential Immune-Related Long Non-coding RNA Prognostic Signature for Ovarian Cancer	34367253

PanX	A Novel Six-Gene Signature for Prognosis Prediction in Ovarian Cancer	33193589
Peng	A prognostic model based on immune-related long noncoding RNAs for patients with epithelial ovarian cancer	35031063
Qi	A nine-gene signature related to tumor microenvironment predicts overall survival with ovarian cancer	32208363
Qingyang	Identifying the Role of Oxidative Stress-Related Genes as Prognostic Biomarkers and Predicting the Response of Immunotherapy and Chemotherapy in Ovarian Cancer.	36561981
Qiu	A Liquid-Liquid Phase Separation-Related Gene Signature as Prognostic Biomarker for Epithelial Ovarian Cancer	34168991
Sheng	Integrative network analysis identifies an immune-based prognostic signature as the determinant for the mesenchymal subtype in epithelial ovarian cancer.	33031300
Su	A novel immune-related prognostic signature in epithelial ovarian carcinoma	33819196
Sun	Identification of a Prognostic Signature Associated With DNA Repair Genes in Ovarian Cancer.	31572446
Wang	Development of a five-gene signature as a novel prognostic marker in ovarian cancer.	30569721
Xiang	Construction of a prognostic signature for serous ovarian cancer based on lactate metabolism-related genes.	36185201
Xiaoqing	Identification of immunity- and ferroptosis-related genes for predicting the prognosis of serous ovarian cancer	35777713
Xin	The cuproptosis-related gene signature serves as a potential prognostic predictor for ovarian cancer using bioinformatics analysis	36267774
Xu	Integration of Transcriptome and Epigenome to Identify and Develop Prognostic Markers for Ovarian Cancer	36081667
Xueyuan	Derivation, Comprehensive Analysis, and Assay Validation of a Pyroptosis-Related lncRNA Prognostic Signature in Patients With Ovarian Cancer	35280739
Xuyao	A signature based on glycosyltransferase genes provides a promising tool for the prediction of prognosis and immunotherapy responsiveness in ovarian cancer	36611197
Yan	Development and Verification of an Autophagy-Related lncRNA Signature to Predict Clinical Outcomes and Therapeutic Responses in Ovarian Cancer	34671615
Yilong	Identification of a novel ferroptosis-related gene signature associated with prognosis, the immune landscape, and biomarkers for immunotherapy in ovarian cancer	36386203
Yang	Integrated analysis of a competing endogenous RNA network reveals an 11-lncRNA prognostic signature in ovarian cancer	33223503
Yuanyuan	Development and Validation of an Immune-Related Prognostic Signature for Ovarian Cancer Based on Weighted Gene Coexpression Network Analysis	33381581
Zeng	Identification of a Gene Signature of Cancer-Associated Fibroblasts to Predict Prognosis in Ovarian Cancer	35873482
Zhao	Exploration of the Immunotyping Landscape and Immune Infiltration-Related Prognostic Markers in Ovarian Cancer Patients.	35880167
Zheng	Identification three LncRNA prognostic signature of ovarian cancer based on genome-wide copy number variation	32000042
